# Investigation of two metabolic engineering approaches for (*R*,*R*)-2,3-butanediol production from glycerol in *Bacillus subtilis*

**DOI:** 10.1186/s13036-022-00320-w

**Published:** 2023-01-10

**Authors:** Nunthaphan Vikromvarasiri, Shuhei Noda, Tomokazu Shirai, Akihiko Kondo

**Affiliations:** 1 RIKEN Center for Sustainable Resource Science, 1‑7‑22 Suehiro‑cho, Tsurumi‑ku, Yokohama, Kanagawa 230‑0045 Japan; 2 Department of Chemical Science and Engineering, Graduate School of Engineering, Kobe University, 1-1 Rokkodai, Nada, Kobe, 657-8501 Japan

**Keywords:** Glycerol, 2,3-Butanediol, *Bacillus subtilis*, Flux balance analysis, OptKnock, Genome-scale metabolic model

## Abstract

**Background:**

Flux Balance Analysis (FBA) is a well-known bioinformatics tool for metabolic engineering design. Previously, we have successfully used single-level FBA to design metabolic fluxes in *Bacillus subtilis* to enhance (*R*,*R*)-2,3-butanediol (2,3-BD) production from glycerol. OptKnock is another powerful technique for devising gene deletion strategies to maximize microbial growth coupling with improved biochemical production. It has never been used in *B. subtilis*. In this study, we aimed to compare the use of single-level FBA and OptKnock for designing enhanced 2,3-BD production from glycerol in *B. subtilis*.

**Results:**

Single-level FBA and OptKnock were used to design metabolic engineering approaches for *B. subtilis* to enhance 2,3-BD production from glycerol. Single-level FBA indicated that deletion of *ackA*, *pta*, *lctE*, and *mmgA* would improve the production of 2,3-BD from glycerol, while OptKnock simulation suggested the deletion of *ackA*, *pta*, *mmgA*, and *zwf*. Consequently, strains LM01 (single-level FBA-based) and MZ02 (OptKnock-based) were constructed, and their capacity to produce 2,3-BD from glycerol was investigated. The deletion of multiple genes did not negatively affect strain growth and glycerol utilization. The highest 2,3-BD production was detected in strain LM01. Strain MZ02 produced 2,3-BD at a similar level as the wild type, indicating that the OptKnock prediction was erroneous. Two-step FBA was performed to examine the reason for the erroneous OptKnock prediction. Interestingly, we newly found that *zwf* gene deletion in strain MZ02 improved lactate production, which has never been reported to date. The predictions of single-level FBA for strain MZ02 were in line with experimental findings.

**Conclusions:**

We showed that single-level FBA is an effective approach for metabolic design and manipulation to enhance 2,3-BD production from glycerol in *B. subtilis*. Further, while this approach predicted the phenotypes of generated strains with high precision, OptKnock prediction was not accurate. We suggest that OptKnock modelling predictions be evaluated by using single-level FBA to ensure the accuracy of metabolic pathway design. Furthermore, the *zwf* gene knockout resulted in the change of metabolic fluxes to enhance the lactate productivity.

**Supplementary Information:**

The online version contains supplementary material available at 10.1186/s13036-022-00320-w.

## Background

For decades, genome-scale metabolic models (GEMs) have been recognized and used as a powerful bioinformatics tool for biological systems. GEMs are computational reconstructions of metabolic networks that can be applied to a wide range of living cells, such as microorganisms, plants, etc. GEMs are constructed based on basic genome annotation and experimental data, and reflect the gene–protein–reaction relationships and the mass, energy, and proton balance of all reactions for the entire set of metabolic genes in an organism [1]. Many software platforms and techniques have been used for GEM reconstruction and metabolic flux prediction for different applications, such as metabolic pathway design for microorganisms of industrial relevance. However, these platforms and techniques have some limitations [2]. Therefore, the application of GEMs for metabolic flux prediction by using mathematical optimization techniques is continuously developed.

Flux Balance Analysis (FBA) is a mathematical optimization technique for the simulation of metabolic fluxes in GEMs using single-level linear programming [3, 4]. FBA requires non-complicated input data for model simulation to resolve the objective function in a steady-state flux distribution using a stoichiometric matrix. It can be used to compare the predicted metabolic flux networks under different environmental conditions to identify suitable conditions for a set of objectives, such as biomass production rate. Specialized software is used to solve large-scale linear programming, to allow FBA calculation of steady-state metabolic fluxes for large models in a relatively short period of time [5]. FBA has been successfully used in the bioengineering of many microbes to improve the production of fermentation compounds for various purposes, such as ethanol production in *Scheffersomyces stipitis* [6], (*R*,*R*)-2,3-butanediol (2,3-BD) production in *Bacillus subtilis* [7], lactate production in *Lactobacillus plantarum* WCSF1 and *Lactobacillus reuteri* JCM 1112 [8], and shikimic acid production in *Escherichia coli* [9].

OptKnock has been developed as a guide for gene deletion strategies to maximize microbial cell (biomass) production together with an improved biochemical production [10]. OptKnock analyzes and defines the metabolic reactions of GEMs that compete with microbial growth reactions coupled with the production of target compounds to suggest reactions for possible deletion. It utilizes a mixed-integer linear programming (MILP) solver, such as Gurobi Optimizer [11] and IBM ILOG CPLEX Optimizer [12]. Burgard et al. [10] used OptKnock to successfully maximize the growth coupling with succinate, lactate, and 1,3-propanediol production in *E. coli*. As another example, Pharkya et al. [13] used OptKnock to design metabolic pathways for the overproduction of amino acids glutamate, serine, aspartate, and alanine in *E. coli.* Further, Feist et al. [14] used OptKnock in *E. coli* GEM (iAF1260 model) to identify patterns for gene deletion solutions, with optimization for 3- to 5-gene deletions, to improve the production of multiple native products in *E. coli* from various types of substrates.

Glycerol is a promising low-cost, renewable, and non-food competitive substrate compared to glucose, which is the main raw material used in the biological fermentation process. The price of high-purity crude glycerol (80–90%) is about 0.00–0.11 USD/kg, whereas the price of glucose is comparatively more expensive (3.37 USD/kg) [15]. Thus, glycerol is a good alternative resource for the bioindustry. It is used for the production of (*R*,*R*)-2,3-butanediol (2,3-BD), a useful chemical compound that has several applications, such as in printing ink, antifreeze agents, foodstuffs, and pharmaceuticals [16]. Furthermore, it can be used as the starting compound for the production of other valuable compounds, such as 1,3-butadiene, acetoin, and diacetyl [17]. Further, the bioproduction of 2,3-BD has a variety of advantages over chemical processes, mainly related to the production costs and environmental concerns [18].

*B. subtilis* is accepted as a “generally recognized as safe” organism that grows in the presence of high levels of fermentation products, such as volatile fatty acids (VFAs), and does not generate endotoxins, unlike gram-negative bacteria, such as *E. coli* [19]. It is an ideal platform microorganism for bioengineering to improve the production of various useful compounds, such as amino acids, lactate, acetate, acetoin, and 2,3-BD.

Previously, we successfully used single-level FBA to design and evaluate the effect of gene knockouts for enhanced 2,3-BD production from glycerol in *B. subtilis* [7]. The predictions of single-level FBA, which focused on “biomass production” as the only objective function, suggested deletion of four genes (namely, *ackA* gene encoding acetate kinase; *pta* gene encoding phosphate acetyltransferase; *lctE* gene encoding l-lactate dehydrogenase; and *mmgA* gene encoding acetyl-CoA acetyltransferase) to enhance the production of 2,3-BD from glycerol in iYO844 GEM. The deletion resulted in a 2,3-BD yield increase by 43.75%, compared to that from the wild-type strain. However, another strategy, OptKnock, has never been applied for improving bio-production in *B. subtilis*.

In the current study, we investigated the use of single-level FBA and OptKnock to manipulate the metabolic flux network of *B. subtilis* for improved 2,3-BD production from glycerol. We used *B. subtilis* GEM (iYO844 model) constructed by Oh et al. [20] to design a prototype of the metabolic pathways in *B. subtilis* for 2,3-BD production from glycerol that showed the most reliable performance with glycerol as a sole carbon source in M9 medium. We then constructed *B. subtilis* deletion mutants following the predictions of single-level FBA and OptKnock, and evaluated changes in the fermentation profile of the resultant strains and wild-type *B. subtilis* strain (W168). We also inspected the accuracy of *in silico* predictions with the fermentation profile.

## Results and discussion

### Single-level FBA and OptKnock designs for the improvement of 2,3-BD production from glycerol in *B. subtilis*

**Fig. 1:**
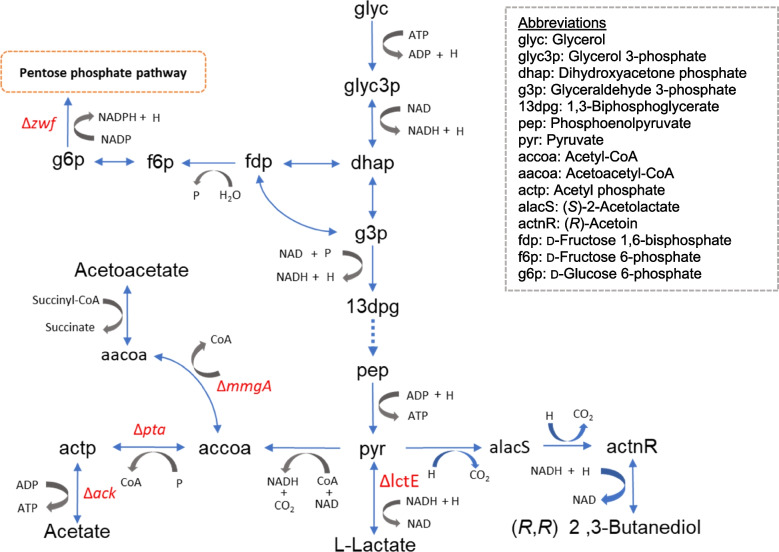
Metabolic pathway of glycerol dissimilation into 2,3-butanediol in *B. subtilis*. Blue arrows indicate metabolic fluxes, and delta symbol (*∆*) followed by gene abbreviation (red font) indicates the genes suggested for knockout by single-level FBA and OptKnock. Gene abbreviations refer to the encoded enzymes, namely: *ack*, acetate kinase; *pta*, phosphate acetyltransferase; *lctE*, l-lactate dehydrogenase; *mmgA*, acetyl-CoA C-acetyltransferase; and *zwf*, glucose 6-phosphate dehydrogenase

We used single-level FBA and OptKnock to redesign metabolic pathways in *B. subtilis* to enhance 2,3-BD production from glycerol*.* We created a metabolic pathway of glycerol dissimilation following flux distribution based on the results of single-level FBA and OptKnock in iYO844 GEM with glycerol as a sole substrate (Fig. 1). Single-level FBA design was based on eliminating the fermentative products competing with 2,3-BD production, as described in our previous study [7]. The only objective function was biomass production. According to single-level FBA, the inhibition of four reactions, namely, ACKr (*ack*), PTAr (*pta*), LDH_L (*lctE*), and ACACT1r (*mmgA*), would enhance 2,3-BD production (Table 1). The removal of ACKr (*ack*) and PTAr (*pta*) reaction was predicted to reduce acetate production. The removal of LDH_L (*lctE*) reaction was predicted to inhibit lactate production. Finally, the removal of ACACT1r (*mmgA*) reaction was predicted to block the production of acetoacetate. According to single-level FBA, inhibition of these four reactions would improve the 2,3-BD production from 0 to 2.54 mmol/gCDW/h (CDW, cell dry weight), compared with the flux in the wild-type strain.

**Table 1:** Flux distribution indicated by different engineering approaches in different strains to enhance 2,3-BD production (mmol/gCDW/h)^a^

Method	Single-level FBA		OptKnock^b^
***B. subtilis* strain**	W168	LM01^c^	W168
Biomass production	0.30	0.28	0.35
Acetate exchange	3.78	0.12	0.15
l-Lactate exchange	2.00	0.00	0.00
(*R*,*R*)-2,3-BD exchange	0.00	2.54	2.10

^a^Glycerol utilization and oxygen consumption were set to 10 mmol/gCDW/h (see Methods)

^b^OptKnock objectives were set for growth coupling with 2,3-BD production. The reactions suggested for gene knockout were: ACKr (*ack*), PTAr (*pta*), ACACT1r (*mmgA*), and G6PDH2r (*zwf*). This informed the construction of MZ02 strain

^c^LM01 was designed to be unable to perform four reactions, namely, ACKr (*ack*), PTAr (*pta*), LDH_L (*lctE*), and ACACT1r (*mmgA*)

OptKnock suggested a different strategy, with the improvement of growth (biomass production) coupling with the improvement of 2,3-BD production set as the objective functions. The modelling suggested the removal of the ACKr (*ack*), PTAr (*pta*), ACACT1r (*mmgA*), and G6PDH2r (*zwf*) reactions. Considering the flux distribution, the biomass production would increase to 0.35 mmol/gCDW/h, with 2,3-BD production at a level of 2.10 mmol/gCDW/h, with the acetate production decreased to 0.15 mmol/gCDW/h (Table 1). Of note, the flux to lactate production became zero in the model, even though the LDH_L (*lctE*) reaction was not removed. To the best of our knowledge, the knockout of the *zwf* gene to enhance 2,3-BD production has not been reported to date. Hence, the analysis suggested that deletion of these genes would impede lactate production and improve 2,3-BD production.

We subsequently constructed mutant strains of *B. subtilis* based on the gene deletions suggested by single-level FBA (strain LM01) and OptKnock (strain MZ02) predictions, and investigated 2,3-BD production during fermentation, with the wild-type strain (W168) as a control. The results of these analyses are described below.

### Effect of gene deletions on the fermentation profile of *B. subtilis*

**Fig. 2:**
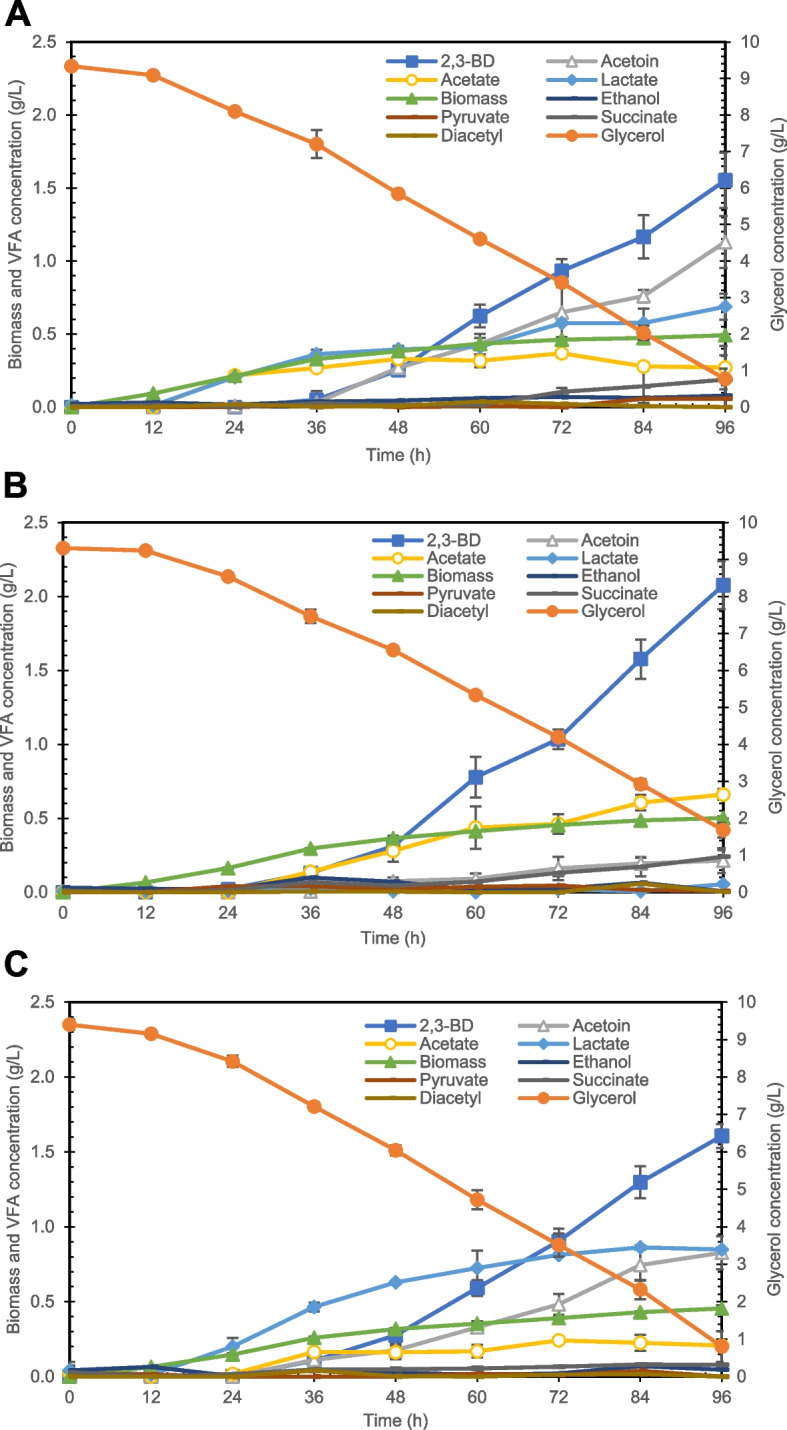
Fermentation profiles, including growth, glycerol utilization, and VFA production of *B. subtilis* strains. Data for the following strains are shown: W168 (**A**), LM01 (**B**), and MZ02 (**C**). The data are presented as the mean ± standard deviation of three independent experiments (*n* = 3)

2,3-BD production from glycerol in *B. subtilis* strains was evaluated using batch fermentation. The deletion of multiple genes in strains LM01 (*ackA*, *pta*, *lctE*, and *mmgA*) and MZ02 (*ackA*, *pta*, *mmgA*, and *zwf*) did not notably negatively affect bacterial growth (Fig. 2). Biomass production during logarithmic (24 h), early stationary (36 h), and late stationary phases (48 h) was similar in all strains. The major fermentation products were acetate, lactate, acetoin, and 2,3-BD, with only a minor production of ethanol, succinate, pyruvate, and diacetyl during the experiment.

We compared the yields of major fermentation products and glycerol utilization by all strains during each growth phase (Table 2). Glycerol utilization in all strains was not affected by the growth phase, and the multiple-gene knockouts did not strongly affect glycerol utilization of the strains. Total biomass production by the W168, LM01, and MZ02 strains was 4.35 ± 0.05, 4.44 ± 0.10, and 4.03 ± 0.19 mmol/L, respectively. The wild-type strain (W168) produced acetate and lactate as the major fermentation products during the logarithmic phase, with these VFAs converted together with glycerol to acetoin and 2,3-BD during the stationary phase.

**Table 2:** Major VFA yields and glycerol utilization during *B. subtilis* growth

**Strain**	**VFA (mol/mol‧glycerol) at 0–24 h**				**Total glycerol utilization (mmol)**
	**Acetate**	**Lactate**	**Acetoin**	**2,3-BD**	
W168	0.27 ± 0.02	0.17 ± 0.00	0.01 ± 0.01	0.00 ± 0.00	13.4 ± 0.37
LM01	0.00 ± 0.00	0.01 ± 0.01	0.00 ± 0.00	0.03 ± 0.05	8.4 ± 0.16
MZ02	0.03 ± 0.03	0.21 ± 0.06	0.00 ± 0.00	0.00 ± 0.00	10.6 ± 1.64
**Strain**	**VFA (mol/mol‧glycerol) at 24–36 h**				**Total glycerol utilization (mmol)**
	**Acetate**	**Lactate**	**Acetoin**	**2,3-BD**	
W168	0.00 ± 0.00	0.00 ± 0.00	0.01 ± 0.01	0.03 ± 0.02	9.7 ± 3.75
LM01	0.12 ± 0.02	0.00 ± 0.00	0.00 ± 0.01	0.05 ± 0.00	11.7 ± 1.79
MZ02	0.09 ± 0.01	0.01 ± 0.01	0.05 ± 0.02	0.05 ± 0.01	13.1 ± 0.45
**Strain**	**VFA (mol/mol‧glycerol) at 36–96 h**				**Total glycerol utilization (mmol)**
	**Acetate**	**Lactate**	**Acetoin**	**2,3-BD**	
W168	0.00 ± 0.00	0.00 ± 0.00	0.12 ± 0.00	0.16 ± 0.01	69.9 ± 1.10
LM01	0.02 ± 0.01	0.00 ± 0.00	0.03 ± 0.01	0.20 ± 0.01	63.8 ± 1.71
MZ02	0.00 ± 0.00	0.00 ± 0.00	0.05 ± 0.00	0.14 ± 0.01	69.6 ± 3.29

The data are presented as the mean ± [error] (*n* = 3)

Considering the deletion of *ackA* and *pta* genes in the LM01 and MZ02 strains, acetate production was inhibited during the logarithmic phase; however, acetate was detected when the growth reached the early stationary phase. This was in agreement with our previous observations [7]. Similar, Fu et al. [21] suggested that the knockout of the *pta* gene does not inhibit acetate production in *B. subtilis*, which might be generated via pathways for acetate or acetyl-phosphate production from other precursors in this bacterium. Indeed, based on metabolic pathways that operate in *B. subtilis* 168 (Kyoto Encyclopedia of Genes and Genomes) [22], acetate can be produced via many pathways not included in the iYO844 model, e.g., one involving the *ydap* gene (BSU04340, EC:1.2.3.3) and *yflL* gene (BSU07640, EC:3.6.1.7), which convert pyruvate to acetyl phosphate, and then acetate. These missing reactions should be analyzed and the information gap filled to improve the iYO844 GEM.

In addition to the above, the LM01 strain lacks the *lctE* and *mmgA* genes. Knocking out *lctE* suppressed lactate production during fermentation. The product of this gene is the main competitor of the 2,3-BD production reaction, competing for NADH consumption [7]. Accordingly, deletion of the *lctE* or *ldh* gene improves 2,3-BD production in different bacteria [21, 23, 24]. Further, improvement of 2,3-BD production by *mmgA* knockout was shown in a previous study [7]. In the current study, the maximum yield of 2,3-BD was 0.28 mol/mol‧glycerol, which was the highest productivity noted among the compared strains. In addition, the LM01 strain produced only a small amount of acetoin, as NADH availability directly drove the metabolic pathway involved towards 2,3-BD production.

**Fig. 3:**
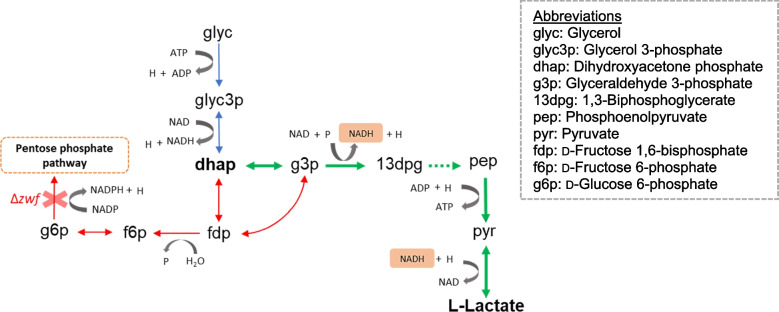
Metabolic fluxes illustrating the enhancing effect of *zwf* gene knockout on the production of lactate. Blue arrows indicate unchanged metabolic fluxes. Red arrows indicate flux decrease relative to wild-type level. Green arrows indicate flux increase relative to wild-type level

In the MZ02 strain, in addition to *ackA* and *pta*, the *mmgA* gene was also deleted to promote 2,3-BD production. However, together with the deletion of the *zwf* gene, this did not improve 2,3-BD production (Table 2). The highest yield of 2,3-BD was 0.19 mol/mol‧glycerol, which was equal to the highest yield of 2,3-BD obtained with the wild-type strain. Surprisingly, lactate was produced during the logarithmic phase at a remarkably high level, compared to that in other strains, with the highest yield of 0.22 mol/mol‧glycerol, in the early stationary phase. In a previous study, lactate yield of *B. subtilis* XZ7 strain was improved by the deletion of the *alsS* gene (encoding α-acetolactate synthase), which inhibited the production of 2,3-BD [25]. Apart from genetic engineering in *B. subtilis*, modifications of some genes in *E. coli* were tested for improved lactate production, e.g., by removing reactions competing for pyruvate, such as the ethanol/acetate metabolic pathway, or precursor reactions converting phosphoenolpyruvate into succinate [26]. To the best of our knowledge, we report here for the first time that the knockout of the *zwf* gene, which is related to glycerol dissimilation to the pentose phosphate pathway, enhances the production of lactate. Considering the single-level FBA predictions, the increase in lactate production might be driven by a decreased metabolic flux from dihydroxyacetone phosphate (dhap) to d-fructose 1,6-bisphosphate (fdp) as a result of the *zwf* gene deletion, with lactate produced by the conversion of dihydroxyacetone phosphate (dhap) to glyceraldehyde 3-phosphate (g3p) (Fig. 3). Hence, the improved flux of glyceraldehyde 3-phosphate dehydrogenase reaction (EC:1.2.1.12; BSU33940) increased the amount of NADH in the system. NADH is consumed in the conversion of pyruvate to lactate and 2,3-BD, which provides a redox balance in the biosynthetic system of *B. subtilis*. And the primary product of fermentation is lactate [27]. Together with the increase of metabolic flux from glyceraldehyde 3-phosphate to pyruvate, this resulted in increased lactate production.

Finally, in the MZ02 strain, acetate and lactate were converted into acetoin and 2,3-BD during the stationary phase, similar to their conversion in the wild-type strain. Since we observed that the gene deletion suggested by OptKnock did not increase 2,3-BD production, we next proceeded to identify the reason for this inaccuracy. Additionally, the change of phenomenon during the fermentation process is over the ability of single-level FBA to analyze. Because FBA can perform only one environmental condition using the linear programming solver. Whereas, the dynamic FBA, which is another cobra toolbox’s function, may be able to analyze this phenomenon.

### Comparison of FBA and OptKnock predictions with experimental data for MZ02 strain

OptKnock focuses on two objective functions, i.e., cell growth and 2,3-BD production, suggesting non-essential reactions and those competing with biomass and 2,3-BD production for removal. The modelling suggested that deletion of four specific reactions (genes) would enhance the biomass and 2,3-BD production in *B. subtilis*, to 0.35 and 2.10 mmol/gCDW/h, respectively (Table 3). However, the experimental data did not confirm these predictions. We hence used a two-step FBA simulation to identify the reason for this discrepancy.

**Table 3:** Comparison of flux distribution predicted by different engineering approaches for different strains (reaction unit: mmol/gCDW/h)^a^

Method	OptKnock^b^	Two-step FBA^c^	Single-level FBA
***B. subtilis* strain**	W168	W168	MZ02^d^
Biomass production	0.35	0.30	0.28
Acetate exchange	0.15	3.12	0.12
l-Lactate exchange	0.00	0.00	4.69
(*R*,*R*)-2,3-BD exchange	2.10	1.35	0.00

^a^Glycerol utilization and oxygen consumption were set to 10 mmol/gCDW/h (see Methods)

^b^OptKnock objectives were set for growth coupling with 2,3-BD production. The reactions suggested for gene knockout were: ACKr (*ack*), PTAr (*pta*), ACACT1r (*mmgA*), G6PDH2r (*zwf*)

^c^Two-step FBA was conducted with the production flux of the target compound maximized with the biomass growth rate, which was fixed at its maximum value. Specifically, the element corresponding to the production of the target compound in a column array containing the objective function coefficients was set to 10^−5^

^d^MZ02 was designed to be unable to perform four reactions, namely, ACKr (*ack*), PTAr (*pta*), ACACT1r (*mmgA*), and G6PDH2r (*zwf*)

Two-step FBA simulates metabolic distribution before gene deletion. We set objective functions similar to those of OptKnock, by maximizing biomass production as the first target and maximizing 2,3-BD production as the second target (Table 3). Biomass production by the wild type predicted by using two-step FBA was the same as that predicted using single-level FBA (0.3 mmol/gCDW/h, Tables 1 and 3). However, the major produced VFAs predicted by two-step FBA (acetate and 2,3-BD) were different from those predicted by single-level FBA (acetate and lactate). This revealed the underlying reason for the inaccurate prediction of OptKnock. Specifically, since acetate and lactate were detected as the major VFA produced in single-level FBA, the reactions for their production were given priority for removal. On the other hand, two-step FBA considered acetate and 2,3-BD as the major products and, consequently, only suggested the reactions for acetate production for removal. LDH_L (*lctE*) reaction was not considered for removal because the initial flux of this reaction was zero. Whereas the flux of *zwf* gene was 0.85 mmol/gCDW/h, which OptKnock selected this reaction as the priority of targeted genes for deletion. This strategy might have led to a bias when assessing biomass and 2,3-BD production, and contributed to the erroneous identification of reactions for removal.

We analyzed the MZ02 strain by single-level FBA, with the biomass production as the only objective function. The predictions corresponded to the experimental data, especially for the logarithmic phase of growth (Tables 2 and 3). The production of acetate was negligible, whereas the production of lactate was higher than that in the wild-type strain. The predictions of single-level FBA for strains W168 and LM01 were also in line with the experimental results (Tables 1 and 2). Hence, we suggest that single-level FBA be accepted as a reliable approach for the design and evaluation of metabolic pathways, as OptKnock was not be applicable to the GEM in the current study. In the case of the current study, a recombinant *B. subtilis* strain should be constructed to allow the conversion of the entire intracellular pyruvate pool to 2,3-BD, not lactate.

Many factors might impact the predictions of OptKnock, such as the degree of completion of the entire metabolic pathways in GEM, the use of solvers for problem function, and so on. Nonetheless, the successful use of OptKnock to optimize biochemical production of native products, especially in the *E. coli* model, was shown in many studies [10, 13, 14]. Overall, we recommend evaluating the OptKnock predictions by using single-level FBA to ensure the precision of the designed metabolic pathway.

## Conclusions

We here investigated the use of OptKnock and single-level FBA simulations for designing metabolic fluxes to enhance the production of 2,3-BD from glycerol in *B. subtilis*. We have previously successfully used single-level FBA to design and evaluate the effect of gene knockouts to enhance 2,3-BD production from glycerol in *B. subtilis* via iYO844 GEM. Single-level FBA suggested the deletion of four genes (*ackA*, *pta*, *lctE*, and *mmgA*; LM01), while OptKnock suggested the deletion of a different gene set (*ackA*, *pta*, *mmgA*, and *zwf*; MZ02). We detected the highest 2,3-BD production in strain LM01, with the 2,3-BD production in MZ02 strain similar to that in the wild-type strain. Consequently, we conducted two-step FBA to examine the reason for the erroneous OptKnock prediction. We observed no flux distribution of lactate after maximizing two objective functions (biomass and 2,3-BD production). Surprisingly, lactate production in strain MZ02 was significantly higher than that in the wild-type strain, which we linked, for the first time, to *zwf* deletion. Predictions of single-level FBA for the MZ02 strain corresponded to the experimental data, as also the predictions of single-level FBA for the LM01 and wild-type strains. Consequently, we propose single-level FBA as one of the reliable methods for the design and evaluation of metabolic pathways of microbial GEMs. We also recommend evaluation of OptKnock predictions by using single-level FBA to confirm the accuracy of the designed metabolic pathway.

## Methods

### *In silico* simulations of single-level FBA, OptKnock, and two-step FBA

In the present study, the iYO844 GEM of *B. subtilis* 168, constructed by Oh et al. [20] and available in BiGG Models [28], was used. Three approaches (single-level FBA, OptKnock, and two-step FBA) were used to simulate metabolic flux distributions. The environmental conditions corresponding to M9 minimal medium for *B. subtilis* were set to the lower bounds of necessary metabolites to allow limitless uptake by the exchange reactions. In all simulation approaches, the maximum uptake rate of glycerol was set at –10 mmol/gCWD/h, with glycerol as the sole carbon source, and the oxygen consumption was limited to –10 mmol/gCWD/h to provide facultative anaerobic conditions. The reactions involving alcohol dehydrogenases (ALCD19_D and ALCD19_L) were removed from GEM, based on Kalantari et al. [29]. The COBRA toolkit [30] was used in MATLAB software 2019b (MathWorks, Inc., Natick, MA) for all simulations of metabolic flux distributions.

Single-level FBA was conducted by using the solver of the commercial GNU Linear Programming Kit (GLPK) package [31]. The solver can be used for large-scale linear programming and mixed-integer programming. Biomass production was set to maximize the target product as a sole objective function. Single-level FBA was used to evaluate the metabolic flux distributions of wild-type and multiple-gene deletion strains. For gene deletions, the minimum and maximum bounds of the fluxes of reactions related to the deletion of target genes were set to zero. Table 4 lists the reactions in the iYO844 model related to the deletion of target genes in *B. subtilis* in the current study. Based on the single-FBA predictions, a multiple-gene deletion mutant of the wild-type strain (W168) was constructed (see below) to increase the production of 2,3-BD (strain LM01; deletion of the ACKr, PTAr, LDH_L, and ACACT1r reactions).

**Table 4:** Correlation of reactions in the iYO844 model with genes and enzymes of *B. subtilis*

Model reaction	Gene^a^	Enzyme	Locus tag	EC number
ACKr	*ackA*	Acetate kinase	BSU29470	2.7.2.1
PTAr	*pta*	Phosphate acetyltransferase	BSU37660	2.3.1.8
LDH_L	*lctE*	l-Lactate dehydrogenase	BSU03050	1.1.1.27
ACACT1r	*mmgA*	Acetyl-CoA C-acetyltransferase	BSU24170	2.3.1.9
G6PDH2r	*zwf*	Glucose 6-phosphate dehydrogenase	BSU23850	1.1.1.49

^a^Data from National Center for Biotechnology Information (NCBI) (accession number: NC_000964.3; accessed 1 April 2022)

OptKnock analysis was performed to determine putative gene deletions in the wild-type strain (W168) for enhanced growth coupling with 2,3-BD production. A solver for MILP problem is required for OptKnock simulation; consequently, the Gurobi Optimizer package [11] was used. The package can be applied to all major problem types, such as linear programming, MILP, quadratic programming, and mixed-integer quadratic programming. The non-essential genes of *B. subtilis* wild type for use in this approach have been determined [32, 33]. The available reactions for OptKnock involving the non-essential *B. subtilis* genes, and not concerning the exchange reactions and transporter reactions in the iYO844 model, were set as the available gene targets for deletion by OptKnock (Additional file 1). Based on the OptKnock predictions, strain MZ02 was constructed (see below; deletion of the ACKr, PTAr, ACACT1r, and G6PDH2r reactions).

To inspect the erroneous predictions of OptKnock, two-step FBA was performed by defining two objective functions for maximization in the wild-type strain, similar to OptKnock simulation. Biomass production was set as the major product and 2,3-BD production was set the minor product. Specifically, the element corresponding to 2,3-BD production in a column array containing the objective function coefficients was set to 10^−5^. The GLPK package was used as a solver. Two-step FBA was used to predict the major fermentation products of the wild-type strain by using two objective functions. The predictions were compared with those of single-level FBA and OptKnock.

Finally, single-level FBA was used to evaluate the metabolic flux distribution in MZ02 strain, with the biomass production set as an objective function, to compare the predicted fermentation profile with the experimental results.

### Strains and plasmid construction

*B. subtilis* wild-type 168 (W168) was obtained from the Microbe Division at the RIKEN BioResource Research Center, Japan. CRISPR-Cas9 system was used for gene knockout in *B. subtilis* [34]. *E. coli* NovaBlue (Novagen, Cambridge, MA) was used for plasmid construction. The strains and plasmids used in the current study are listed in Table 5. Plasmid construction for gene knockout is described elsewhere [7].

**Table 5:** Strains and plasmids used in the current study

Strain or plasmid	Genotype or relevant characteristic	Reference
**Strains**		
* E. coli* Nova blue	*endA1 hsdR17*(r_*K12*_^−^m_*K12*_^+^) *supE44 thi-I gyrA96 relA1 lac recA1*/F’[proAB + lacIq ZΔM15::Tn*10*(Tetr)]	Novagen
* B. subtilis* W168	*trpC2*	JMC^a^
* B. subtilis* LM01	*trpC2 ∆ackA ∆pta ∆lctE ∆mmgA*	[7]
* B. subtilis* MZ02	*trpC2 ∆ackA ∆pta ∆mmgA ∆zwf*	This study
**Plasmids**		
pJOE8999	Kan^R^, P_*manPA*_-*cas9*, P_vanP*_, *lacPOZ’*-gRNA, *oop ter*, T7P, *repE194*^*ts*^, pUC*ori*	[34]
pJOE_ackA	pJOE8999 derivative harboring 20-nt spacer for *ackA* gene targeting and homologous arms for fusion	[7]
pJOE_pta	pJOE8999 derivative harboring 20-nt spacer for *pta* gene targeting and homologous arms for fusion	[7]
pJOE_mmgA	pJOE8999 derivative harboring 20-nt spacer for *mmgA* gene targeting and homologous arms for fusion	[7]
pJOE_zwf	pJOE8999 derivative harboring 20-nt spacer for *zwf* gene targeting and homologous arms for fusion	This study

^a^Microbe Division/Japan Collection of Microorganisms (JMC), RIKEN BioResource Research Center

### Culture media and fermentation conditions

Luria–Bertani (LB) liquid medium, containing (per liter) 10.0 g tryptone, 5.0 g yeast extract, and 10.0 g sodium chloride, was used for the maintenance of all strains and plasmid construction. All strains were preserved at –80 °C and re-activated on LB agar plates (LB medium supplemented with 1.5% (w/v) bacto-agar). M9 medium for *B. subtilis* [35], which contained glycerol as the sole carbon source (10 g/L), was used in experiments evaluating the production of 2,3-BD. The fermentation process was started by inoculating *B. subtilis* colonies in M9 medium (5 mL) and incubating at 37 °C, 180 rpm, overnight. After that, 1% (v/v) of overnight culture was placed in M9 medium and incubated at 37 °C, 180 rpm, for 18 h. Finally, 0.5% (v/v) of the culture was used as the seed culture for the experiment. The cells were grown under micro-aerobic conditions in 120 mL of M9 medium, incubated at 37 °C and 100 rpm for 96 h. The liquid medium was periodically sampled and bacterial growth, and the concentration of glycerol and fermentation products (acetate, lactate, pyruvate, succinate, diacetyl, acetoin, 2,3-BD, and ethanol) determined, as described below.

### Analytical methods

The growth of *B. subtilis* cells was determined by monitoring the culture optical density at 600 nm (OD600) using a UVmini-1240 spectrophotometer (Shimadzu, Kyoto, Japan). CDW was calculated based on the OD600 readings by using the 1 g CDW/L per OD600 ratio of 0.325 [36]. The biomass formula of a microbial cell is C_5_H_7_O_2_N, with the molecular weight of 113 g/mol [37], and was used to convert CDW into the moles of microbial biomass. The concentration of glycerol and fermentation products was analyzed by using high-performance liquid chromatography instrument (Shimadzu, Japan) equipped with a UV/RI detector and an Aminex HPX-87H column (300 × 7.8 mm., Bio-Rad, USA). The chromatography system was operated at 55 °C, with 10-μL sample injection. The flow rate of the mobile phase (5 mM H_2_SO_4_) was set at 0.5 mL/min. The chromatography data were compared to the standard curves of glycerol, acetate, lactate, pyruvate, succinate, diacetyl, acetoin, 2,3-BD, and ethanol.

## Supplementary Information

**Additional file 1.** Non-essential reactions in iYO844 GEM available for OptKnock prediction. 

## Data Availability

All data generated or analyzed during this study are included in this published article and its supplementary information files.

## Abbreviations

2,3-BD: (*R*,*R*)-2,3-Butanediol
CDW: Cell dry weight
FBA: Flux balance analysis
GEM: Genome-scale metabolic model
LB: Luria–Bertani
MILP: Mixed-integer linear programming
VFA: Volatile fatty acid
