# Supplementary material for: Investigation of two metabolic engineering approaches for (R,R)-2,3-butanediol production from glycerol in Bacillus subtilis
Source: J Biol Eng. 2023 Jan 10;17:3. doi: 10.1186/s13036-022-00320-w (PMC9830791; doi:10.1186/s13036-022-00320-w)
Supplement: Supplementary file 1 — Additional file 1. Non-essential reactions in iYO844 GEM available for OptKnock prediction. [file 13036_2022_320_MOESM1_ESM.docx]

**Additional files**

**Additional file 1** Non-essential reactions in iYO844 GEM available for OptKnock prediction

'FBP', 'ACACT1r', 'ACGK', 'ACKr', 'ACLDC', 'ACONT', 'ACOTA', 'APTA1i', 'AGPR', 'ATPM', 'ASPK', 'ASPTA', 'DDPA', 'BTDD_RR', 'CS', 'CYOR3m', 'CYOO3', 'CHORS', 'DHAD1', 'DHAD2', 'GAPD', 'DHQTi', 'DHQS', 'CO3E', 'FUM', 'G6PDH2r', 'GLNS', 'GLYK', 'HSDy', 'SK', 'HSST', 'ICDHyr', 'ILEDHr', 'IPMD', 'IPPS', 'IPPMIa', 'IPPMIb', 'KARA1', 'KARA2', 'LLEUDr', 'LEUTA', 'OMCDC', 'LDH_L', 'PGL', 'MDH', 'ORNTAC_1', 'NDPK3', 'NDPK2', 'PGI', 'PTAr', 'RPE', 'SHK3Dr', 'SHKK', 'OCOAT1', 'PSCVT', 'PSERT', 'PYK', 'SUCD1', 'SUCD5_BS', 'SUCOAS', 'TALA', 'THRS', 'ACLS', 'ACHBS', 'DHAD3', 'PSP_L', 'PGCD', 'GND'
